# Elevated serum TLR4 level as a potential marker for postsurgical chronic pain in pediatric patients with different approaches to analgesia

**DOI:** 10.3389/fmed.2022.897533

**Published:** 2022-08-17

**Authors:** Yaroslav Semkovych, Dmytro Dmytriiev

**Affiliations:** ^1^Department of Children Diseases of Postgraduate Medical Education Faculty, Ivano-Frankivsk National Medical University, Ivano-Frankivsk, Ukraine; ^2^Department of Anesthesiology and Intensive Care, Vinnytsia National Pirogov Memorial Medical University, Vinnytsya, Ukraine

**Keywords:** pain, chronic pain, Toll-like receptor 4, children, regional anesthesia

## Abstract

**Introduction:**

The perioperative period of any surgery is accompanied by immune suppression. The level of Toll-like receptor 4 (TLR4) is known to increase in inflammation and after nerve injury and contributes to the development of neuropathic pain. The interaction of TLRs in response to the effect of opioids results in paradoxical hyperalgesia. Regional anesthesia techniques are the standard of care for perioperative pain management in children.

**Aim:**

The aim of the study was to determine and evaluate the indicators of TLR4 for different methods of pain relief in anesthetic management of hernia repair in children and their effect on pain chronification.

**Materials and methods:**

There were examined 60 children with inguinal hernia during 2020–2022. Children were divided into 3 groups: Group I included 20 children who underwent surgery under general anesthesia using the block of the anterior abdominal wall—transversalis fascia plane block (TFPB), combined with the quadratus lumborum block (QLB-4) *via* a single intramuscular injection; Group II included 20 children who underwent surgery under general anesthesia using the TFPB; Group III comprised 20 children who underwent surgery under general anesthesia using opioid analgesics. The levels of TLR4 were evaluated at a discharge from the hospital, 3 and 6 months after surgery.

**Results:**

There was no difference in age and body weight among all groups. In Group II, boys prevailed. In Group III, the length of hospital stay was the longest (3.28 ± 0.24 days, *p* < 0.05, *t* = 4.09) as compared to children of Group II and Group I (3.0 ± 0.30 (*p* < 0.05, *t* = 2.647) and 2.1 ± 0.16 days, respectively). While staying in the surgical department, children of Group III demonstrated significantly higher FLACC and VAS scores. The prevalence of chronic pain was the highest among children of Group III (35%) as compared to those in Group II and Group I (20 and 15%, respectively). The highest increase in the level of TLR4 was found in the group of opioid analgesia on the third and sixth months after surgery (68.86 + 10.31 pg/ml and 143.15 + 18.77 pg/ml (*p* < 0.05, *t* = 6.33), respectively) as compared to patients who received regional anesthesia.

**Conclusions:**

There were confirmed the following advantages of the transversalis fascia plane block combined with the quadratus lumborum block (QLB + TFPB) *via* a single intramuscular injection: ease of use; adequate perioperative pain control as evidenced by the FLACC and VAS pain assessment scales; reduced perioperative use of opioid analgesics; shortening the length of hospital stay.

## Introduction

The lack of reliable biomarkers to demonstrate the efficacy of therapy and predict disease progression is one of key challenges in pain management (1, 2).

The innate immune system is the body's first line of defense that responds to pathogens and causes pain response (3, 4). The perioperative period of any surgery is accompanied by immune suppression that results from the interaction of several factors, including medications used for post-operative pain control, opioids in particular. The risk of post-operative infections and sepsis increases (5–7).

The innate immune system is activated by pathogens or damage-associated molecular patterns (DAMPs) through Toll-like receptors (TLRs) and Nod-like receptors (NLRs) (8). The level of TLR4 is known to increase in inflammation and after nerve injury and contributes to the development of neuropathic pain (9). TLR2 and TLR3 have been found to play a crucial role in neuropathic pain through the activation of spinal cord glial cells (10, 11).

The innate immune system recognizes ligands *via* several classes of receptors, known as pattern recognition receptors (PRRs). The TLR family of receptors were the earliest PRRs discovered to play an important role in the innate immune response by inducing transcription of pro-inflammatory cytokines interleukin 1 (IL-1), IL-6, and IL-8 in human monocytes (12–14).

Understanding the universal yet unique effect of TLRs on the development and maintenance of persistent (chronic) pain holds promise to improve pain management. Neuropathic and dysfunctional pain are considered as the result of increased sensory signals in the peripheral and central nervous systems (15, 16).

Surgical stress can activate the sympathetic nervous system and the hypothalamic-pituitary-adrenal axis to induce the neuroendocrine response (17) that suppresses T-cell responses (18). There is evidence that morphine and other opioid drugs lead to neuroinflammatory responses, partly mediated *via* glial TLR4 expression (19). Morphine binds to a hydrophobic pocket of myeloid differentiation protein 2 (MD-2) (like the lipid A portion of lipopolysaccharides) and induces TLR4 oligomerization, resulting in the release of IL-1β, tumor necrosis factor alpha (TNF-α), and nitric oxide. The interaction of TLRs in response to the effect of opioids results in paradoxical hyperalgesia that is increased pain sensitization caused by exposure to opioids.

To date, there is no promising therapy for neuropathic pain. Current treatment regimens include tricyclic antidepressants, ion channel modulators (gabapentin, pregabalin, carbamazepine, lidocaine) and some anticonvulsants. However, this arsenal of drugs is often ineffective and demonstrates side effects (20, 21).

Regional anesthesia (RA) techniques are the most valuable and safest methods to treat perioperative pain in children. Notable progress has been made in the development of RA in children over the past few years, including the availability of information on safety, nomenclature, and ultrasound prioritization (22, 23).

The aim of the study was to assess the severity of inflammatory response in children by means of TLR4 indicators while using various regional anesthesia techniques in anesthetic management of hernia repair and their effect on the development of chronic pain syndrome.

## Materials and methods

The study involved 60 (35 boys and 25 girls) children at the age of 7–18 years with inguinal hernia, who were hospitalized to the Pediatric Surgery Clinic of National Pirogov Memorial Medical University, Vinnytsya, Ukraine and the Surgical Department of the Ivano-Frankivsk Regional Children's Clinical Hospital, Ivano-Frankivsk, Ukraine in 2020–2022. The age of 7 is considered the lower limit when a child is capable of self-reporting pain. Inclusion criteria included children at the age of 7–18 years with inguinal hernia, ASA grades I-II, with the mandatory parental consent to involve their child in clinical research. Exclusion criteria included children <7 years of age; those with ASA grade III or higher, mental disorders, neoplasms or tumors, acute or inflammatory processes of any etiology and localization, sepsis, shock; those who previously underwent surgery on the lower abdomen; those who experienced pain for 6 months prior to surgery; those who refused to participate in the research; children whose parents refused to give consent and children who gave no consent.

**Figure 1:**
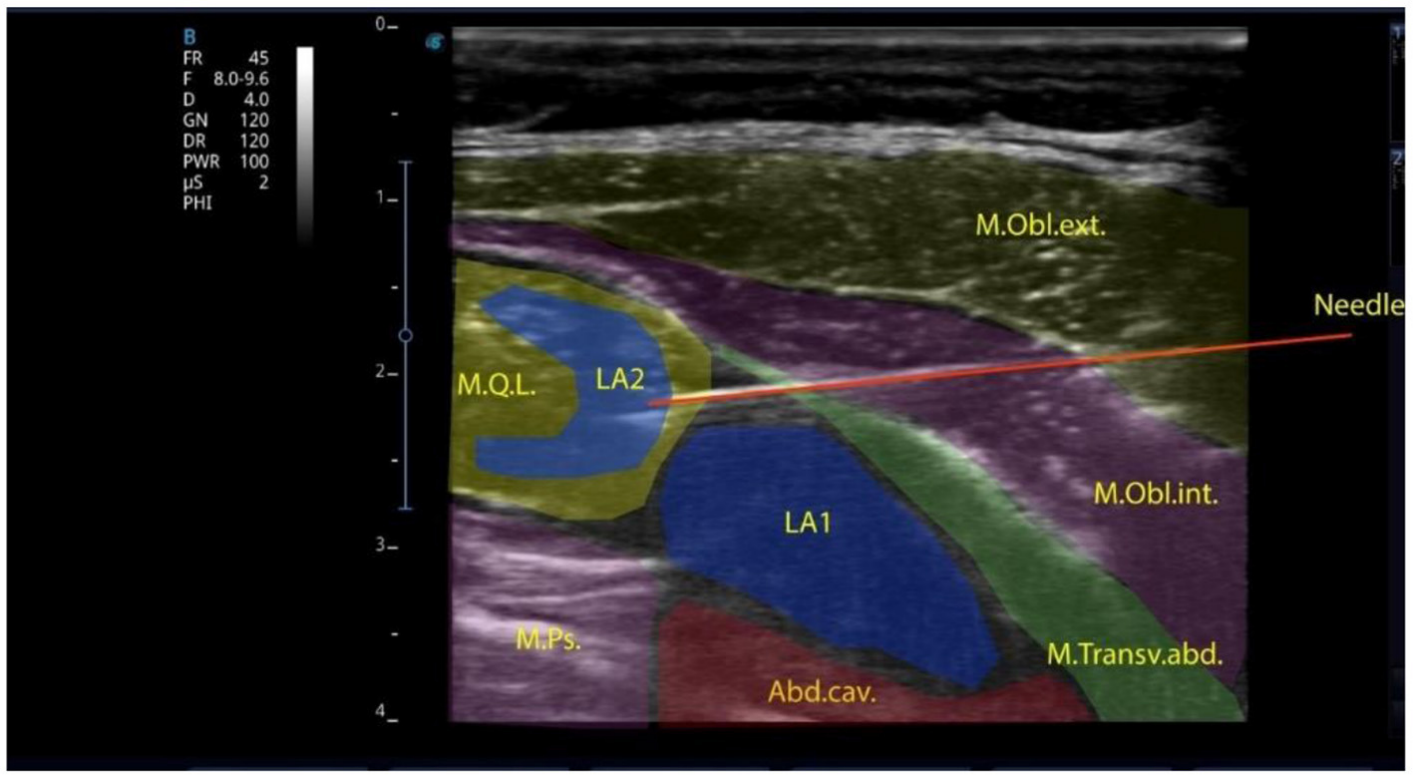
Local anesthetic injected into the transversalis fascia plane in the lumbar region and the quadratus lumborum muscle intramuscularly.

All patients were divided into 3 groups depending on the type of anesthesia. Group I comprised 20 children who underwent surgery under general anesthesia using the block of the anterior abdominal wall—transversalis fascia plane block (TFPB), combined with the quadratus lumborum block (QLB-4) *via* a single intramuscular injection (Figure 1).

The block was performed with a linear high-frequency (7–12 MHz) ultrasonic transducer, the T-Lite ultrasound system by Sonoscanner (France). The transducer was positioned transversely above the abdominal wall muscles. The transverse abdominal muscle, the external and internal abdominal oblique muscles were identified; then, the transducer was sled posteriorly to visualize the quadratus lumborum muscle, the transverse fascia and the preperitoneal space. An 88-mm-long needle was inserted in-plane into the transverse fascia, with anterolateral to posteromedial orientation. To facilitate better needle visualization, there can be performed trial hydrodissection of the fascia with 1–2 mL of saline solution. A syringe with local anesthetic (bupivacaine 0.25% solution is used as the best-choice medication) is attached and the administration of the preparations at a rate 0.2–0.3 ml/kg starts. After anesthetic administration, under ultrasound guidance, the needle, slightly changing its angle, is advanced directly to the quadratus lumborum muscle and a repeated dose of 0.25% bupivacaine (at a rate of 0.2–0.3 ml/kg) is injected. Ultrasound-guided QLB is one of the interfascial plane blocks to provide analgesia during abdominal surgery in adults and children (24). To date, the mechanisms of analgesia development after the QLB have not been sufficiently studied. Visceral analgesia results from the spread of local anesthetic to the abdominal ganglion or sympathetic trunks of the splanchnic nerves, as in the paravertebral blocks. Local anesthetic spreads cranially to the T7-T10 segments (25). The analgesic effect of the QLB may result from the block of mechanoreceptors (Ruffini and Vater-Pacini corpuscles) and nociceptors sensitive to local anesthetics (26). These receptors potentiate sensitivity to acute pain and formation of chronic pain. The QLB is indicated in cases when the dual pain components (somatic and visceral) should be affected: cesarean section (27), gynecologic surgeries (e.g., hysterectomy) (28), small bowel resection (29), large bowel resection (30), nephrectomy, colostomy closure, appendectomy (31), gastrectomy, hernia repair (32).

Group II included 20 children who underwent surgery under general anesthesia using the TFPB. The TFPB is a truncal block that targets the L1 nerve branches, namely the ilioinguinal and iliohypogastric nerves, where they emerge from the lateral border of the psoas major muscle, inferior to the 12th rib. It is used during surgeries on inguinal hernia, trephine biopsy of the iliac spine, chronic neuropathic pain in adults (33).

Group III comprised 20 children who underwent surgery under general anesthesia using morphine.

To determine the levels of TLR4 as an inflammatory marker, all patients underwent venous blood sampling at a discharge from the hospital, 3 and 6 months after surgery. The collected blood was placed in EDTA vacutainers for further plasma extraction by centrifugation. The resulting plasma was frozen and stored at −800°C until further study. The level of TLR4 was determined by means of enzyme-linked immunosorbent assay (ELISA) kit from Elabscience, Lot TM5TMWVDI (USA), according to the manufacturer instructions. The results obtained were determined by the absorption level of the studied samples on the Microtiter plate reader “HumaReader” (Germany) at a wavelength of 450 nm. The minimum possible concentration of determination is 1 pg/ml.

All clinical and laboratory studies were conducted in accordance with the WMA Declaration of Helsinki “Ethical Principles for Medical Research Involving Human Subjects.” Prior to starting the study, each subject (parents/guardians) signed an informed consent for the study. The manuscript was approved by the Ethics Committee of Communal Non-Profit Enterprise “Ivano-Frankivsk Regional Children's Clinical Hospital of Ivano-Frankivsk Regional Council” as evidenced by an excerpt from the minute of the Committee meeting No. 2 dated March 15, 2022.

The analysis of the results obtained, and statistical data processing were carried out using the Statistica 6.0 software package for Windows and the licensed version of BioStat. The differences between the indicators obtained were considered statistically significant at p <0.05. The proportions were statistically compared by using a z-test.

The data from continuous quantitative indicators, which obeyed the law of normal distribution were compared with the use of Student's t-criterion for independent or paired samples. To evaluate and compare different TLR4 parameters, we used the receiver operating characteristic (ROC) curve, which is a graphical representation of sensitivity on the ordinate axis and specificity in the abscissa, and the area under the curve (AUC), which demonstrates the accuracy of the indicator.

## Results

The assessment of children's age, body weight, and gender found that there was no difference in age and body weight, that indicated a representative sample. The assessment of gender (not biological sex) revealed no difference between boys and girls in Group I and III, whereas, in Group II, there was found a significant male predominance (Table 1).

**Table 1:** Distribution of patients by age, body weight, and gender.

**Indicator**	**Group I**	**Group II**	**Group III**
	***n* = 20**	***n =* 20**	***n* = 20**
	**M ±m**	**M ±m**	**M ±m**
Age, years	9.78 ± 0.23	9.12 ± 0.56	9.78 ± 0.45
Body weight, kg	36.6 ± 1.61	35.11 ± 1.19	34.09 ± 1.34
Boys, %	51.4 ± 0.84%	56.21 ± 2.31%	53.42 ± 1.31%
Girls, %	48.6 ± 1.24%	43.9 ± 1.17%^*^ (4.754)	46.58 ± 1.27%

* *A significant difference between boys and girls in corresponding groups (p <0.05)*.

According to the analysis of the length of hospital stay in the surgical department, children, who received conventional anesthesia management, stayed at the hospital much longer as compared to those who received RA (3.28 ± 0.24 days in Group III vs. 3.0 ± 0.30 and 2.1 ± 0.16 days in Group II and Group I, respectively, *p* < 0.05, *t* = 2.647—Group II, *t* = 4.09—Group III). It is worth mentioning that children, who received the QLB combined with the TFPB, were discharged home on the 2.1 ± 0.16th day, while those, who received the TFPB only, were discharged from the hospital on the 3.0 ± 0.30th day (*p* < 0.05), which indicated the efficacy of the proposed combined single-injection block (Table 2).

**Table 2:** Length of hospital stay in the surgical department.

**Indicator**	**Group I**	**Group II**	**Group III**
	***n* = 20**	***n* = 20**	***n* = 20**
	**M ±m**	**M ±m**	**M ±m**
Length of stay in the department	2.1 ± 0.16	3.0 ± 0.30^*^	3.28 ± 0.24^*^
		(*t* = 2.647)	(*t* = 4.09)

* *A significant difference as compared to Group I (p <0.05)*.

**Figure 2:**
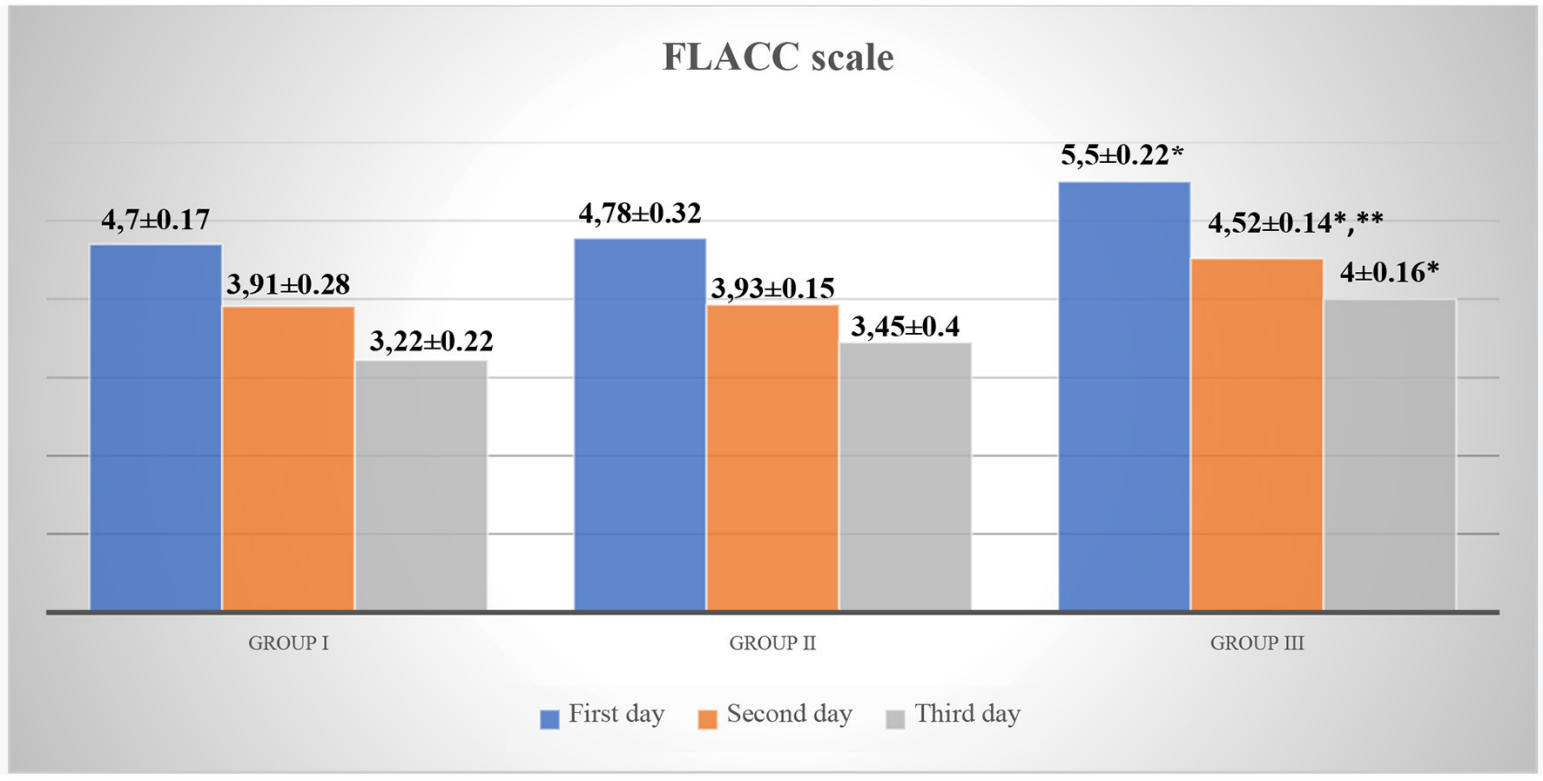
FLACC scale for acute pain assessment in patients. *p < 0.05—a significant difference between children in Group III and Group I. **p < 0.05—a significant difference between children in Group III and Group II.

**Figure 3:**
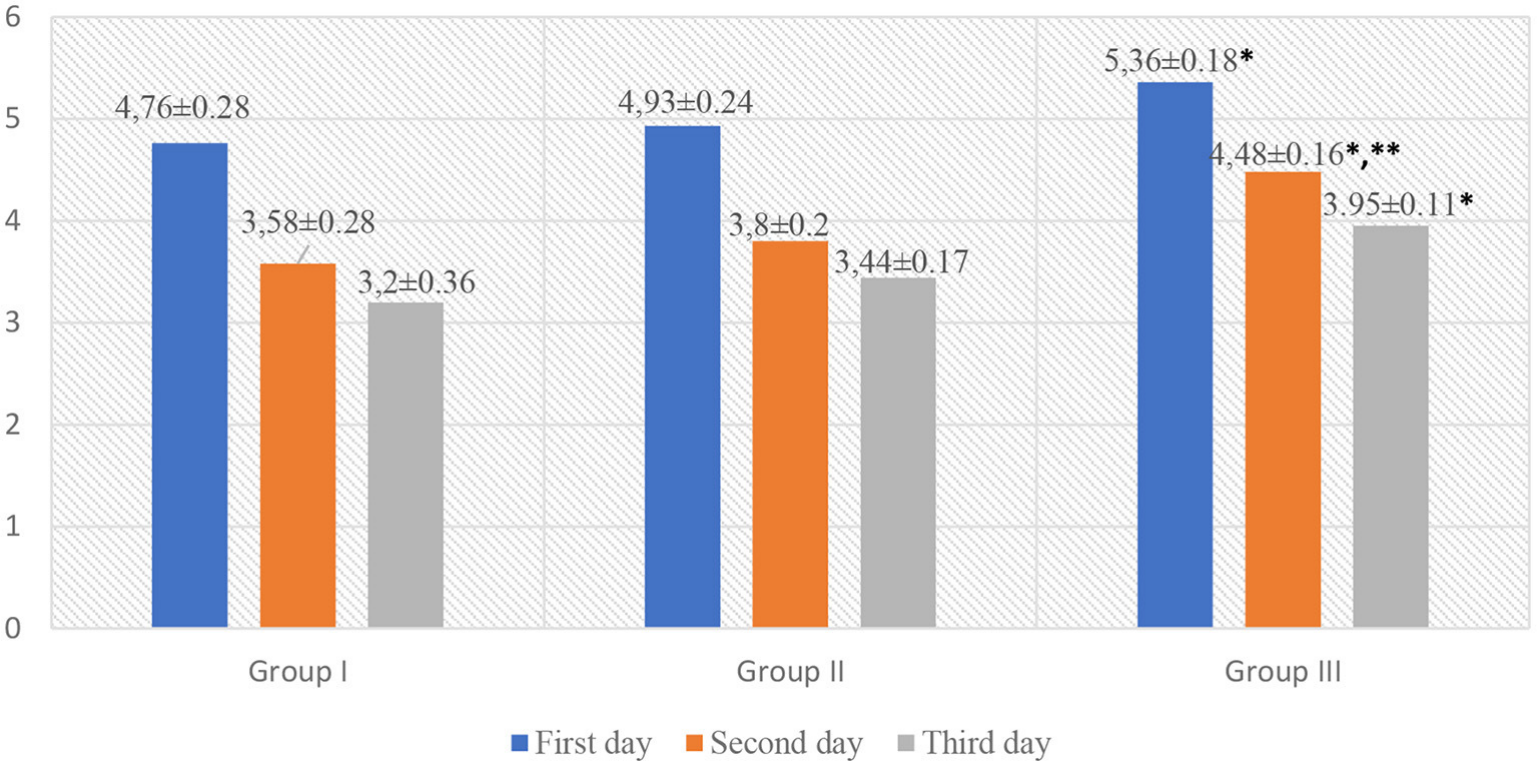
VAS scale for acute pain assessment in patients. *p < 0.05—a significant difference between children in Group III and Group I. **p < 0.05—a significant difference between children in Group III and Group II.

The analysis of the indicators of scales for assessing acute pain in children revealed that children in Group III, while staying in the surgical department, had significantly higher Face, Legs, Activity, Cry, Consolability scale (FLACC) and Visual Analog Scale (VAS) scores as compared to those in Group I (*p* < 0.05, *t* = 2.88). However, on the 2nd day of hospital stay, pain intensity was higher in Group III (FLACC −4.52 ± 0.14, VAS −4.48 ± 0.16) as compared to Group I (FLACC −3.91 ± 0.28, *p* < 0.05, t1 = 2.1, VAS −3.58 ± 0.28, *p* < 0.05, t1 = 2.79) and Group II (FLACC −3.93 ± 0.15, *p* < 0.05, t2 = 2.875 VAS −3.8 ± 0.2, *p* < 0.05, t2 = 2.655). On the 3nd day of hospital stay, pain intensity was higher in Group III (FLACC −4.0 ± 0.16, VAS −3.95 ± 0.11) as compared to Group I (FLACC −3.22 ± 0.22, *p* < 0.05, *t* = 2.867, VAS −3.2 ± 0.33, *p* < 0.05, *t* = 2.156) and Group II (FLACC −3.45 ± 0.4, VAS −3.44 ± 0.17) (Table 3, Figures 2, 3).

**Table 3:** Scales for assessing acute pain in patients.

**Indicator**		**Group I**	**Group II**	**Group III**
		***n* = 20**	***n* = 20**	***n* = 20**
		**M ±m**	**M ±m**	**M ±m**
FLACC	1st day	4.7 ± 0.17	4.78 ± 0.32	5.5 ± 0.22*
				*t* = 2.88
	2nd day	3.91 ± 0.28	3.93 ± 0.15	4.52 ± 0.14^*^
				^**^t1 = 2.1
				*t*2 = 2.875
	3rd day	3.22 ± 0.22	3.45 ± 0.40	4.0 ± 0.16*
				*t* = 2.867
VAS	1st day	4.76 ± 0.28	4.93 ± 0.24	5.36 ± 0.18*
				*t* = 2.49
	2nd day	3.58 ± 0.28	3.8 ± 0.2	4.48 ± 0.16^*^
				^**^t1 = 2.79
				*t*2 = 2.655
	3rd day	3.2 ± 0.33	3.44 ± 0.17	3.95 ± 0.11*
				*t* = 2.156

^*^p <0.05—a significant difference between children in Group III and Group I.

^**^p <0.05—a significant difference between children in Group III and Group II.

Graphic representation of pain assessment according to the FLACC/VAS scales at different study periods among patients of all groups is shown in Figures 2, 3.

According to the analysis of questionnaires for acute pain assessment in children (DN4 questionnaire, LANSS Pain Scale), the prevalence of chronic pain was the greatest in children of Group III (35%) as compared to those in Group II and Group I (20 and 15%, respectively), which again confirmed the efficacy of the QLB combined with the TFPB for prevention and treatment of acute pain, as well as development of chronic pain syndrome (Table 4).

**Table 4:** Scales for chronic pain assessment in patients.

**Indicator**		**Group I**	**Group II**	**Group III**
		***n* = 3**	***n* = 4**	***n* = 7**
		**M ±m**	**M ±m**	**M ±m**
DN-4	3 months	4.5 ± 0.5	4.6 ± 0.24	5.14 ± 0.26
	6 months	4.33 ± 0.33	4.2 ± 0.2	4.78 ± 0.05
LANSS	3 months	12.6 ± 0.33	13.4 ± 0.024	14.11 ± 0.53
	6 months	12.3 ± 0.33	12.8 ± 0.2	13.14 ± 0.14

This study examined a biomarker for inflammation, namely TLR4 in pediatric patients who underwent herniotomy and assessed the level of inflammatory response depending on analgesic technique. The levels of TLR4 at certain post-operative periods are given in Table 5.

**Table 5:** Changes in TLR4 levels among patients of all groups at different study periods.

**Serum TLR4 level**	**Determination of TLR4 level**		
	**At a discharge from the hospital**	**3 months after surgery**	**6 months after surgery**
Group I (*n* = 20)	15.27 ± 3.31 pg/ml*	39.67 ± 7.18 pg/ml	48.18 ± 7.62 pg/ml
*p*		<0.05 *t* = 3,086	<0.05 *t* = 3.96
Group II (*n* = 20)	18.34 ± 2.84 pg/ml*	54.26 ± 9.12 pg/ml	115.57 ± 16.32 pg/ml
*p*		<0.05 *t* = 3.76	<0.05 *t* = 5.87
Group III (*n* = 20)	20.78 ± 4.58 pg/ml*	68.86 ± 10.31 pg/ml	143.15 ± 18.77 pg/ml
*p*		<0.05 *t* = 4.26	<0.05 *t* = 6.33

*A significant difference in TLR4 levels after surgery.

In Group I, there was found an increase in serum level of TLR4 by 2.6 times 3 months after surgery −39.67 + 7.18 pg/ml vs. 15.27 + 3.31 pg/ml at a discharge from the hospital (*p* < 0.05, *t* = 3,086), respectively, and by 3.15 times 6 months after surgery −48.18 + 7.62 pg/ml vs. 15.27 + 3.31 pg/ml at a discharge from the hospital (*p* < 0.05, *t* = 3.96), respectively.

A similar dynamics of increase in serum level of TLR4 was observed in Group II; however, it was more pronounced. Three months after surgery, TLR4 level was 2.96 times higher than that at a discharge from the hospital −54.26 + 9.12 pg/ml vs. 18.34 ± 2.84 pg/ml (*p* < 0.05, *t* = 3.76), respectively; 6 months after surgery, it was 6.3 times higher −115.57 + 16.32 pg/ml vs. 18.34 ± 2.84 pg/ml (*p* < 0.05, *t* = 5.87), respectively.

An even more pronounced increase in TLR4 level was observed in patients of Group III. Three months after surgery, the level of cytokine was 3.31 times higher than that at a discharge from the hospital −68.86 + 10.31 pg/ml vs. 20.78 ± 4.58 pg/ml (*p* < 0.05, *t* =4 .26), respectively; 6 months after surgery, the increase in cytokine level exceeded the value of TLR4 at a discharge from the hospital by 6.9 times −143.15 + 18.77 pg/ml vs. 20.78 ± 4.58 pg/ml (*p* < 0.05, *t* = 6.33), respectively.

To assess and compare the levels of TLR4 in all study groups, an ROC curve was constructed to determine the sensitivity and specificity of the data obtained on the chosen method of anesthesia for hernia repair in children.

**Figure 4:**
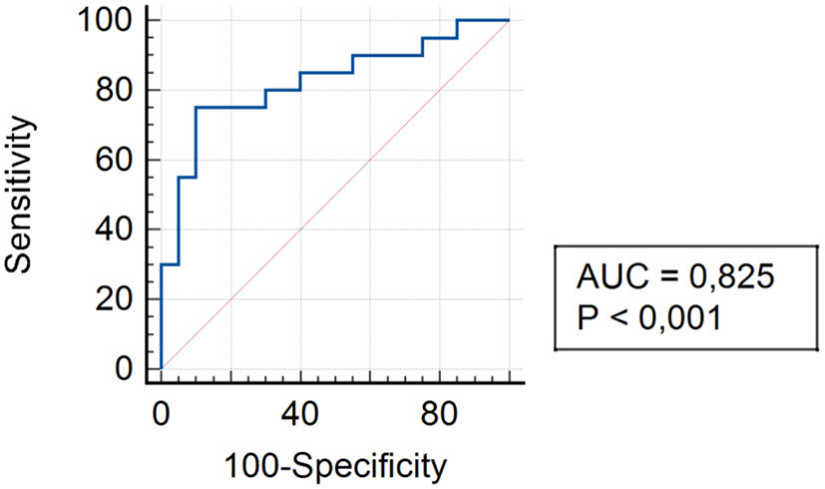
ROC curve for TLR4 expression in patients of Group I 3 and 6 months after surgery.

The analysis of assessing the specificity and sensitivity of serum TLR4 levels in children of Group I showed that the AUC was 0.825 [95% confidence interval (CI) 0.672–0.925]. The cut-off point was 45.4 pg/ml, where the sensitivity was 75.0%, specificity was 90.0% (Figure 4).

**Figure 5:**
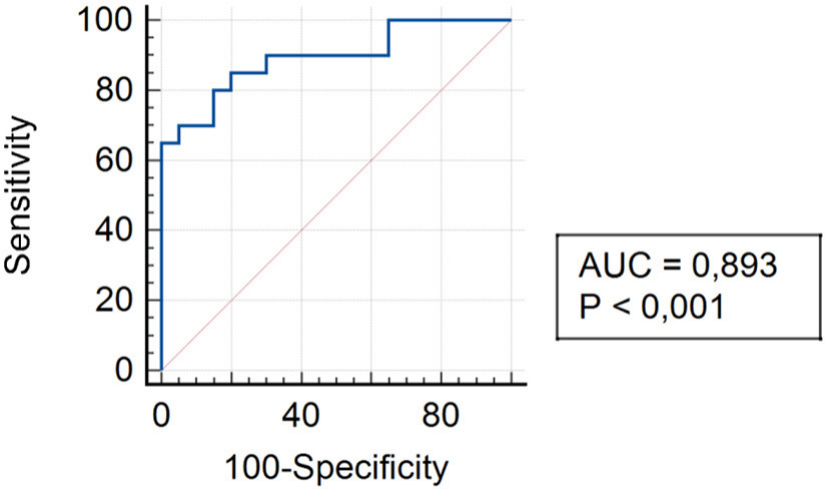
ROC curve for TLR4 expression in patients of Group II 3 and 6 months after surgery.

The analysis of assessing the specificity and sensitivity of serum TLR4 levels in children of Group II showed that the AUC was 0.893 (95% CI 0.754–0.968). The cut-off point was 75.3 pg/ml, where the sensitivity was 85.0% and specificity was 80.0% (Figure 5).

**Figure 6:**
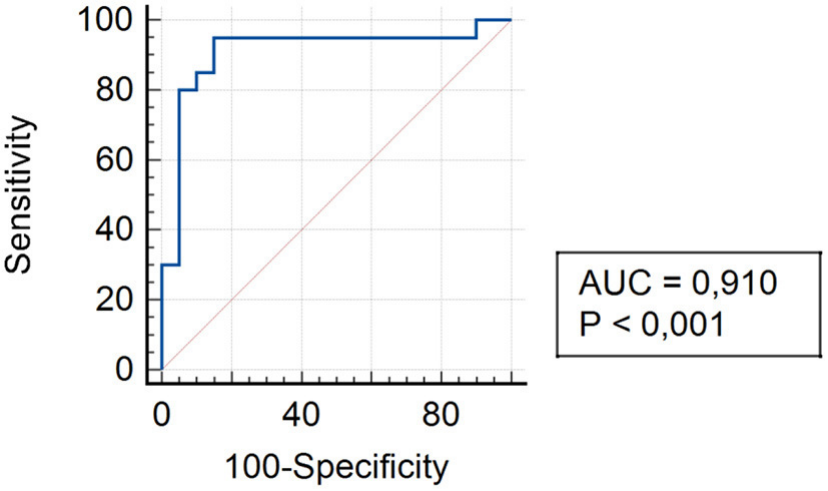
ROC curve for TLR4 expression in patients of Group III 3 and 6 months after surgery.

The analysis of assessing the specificity and sensitivity of serum TLR4 levels in children of Group III showed that the AUC was 0.910 (95% CI 0.776–0.977). The cut-off point was 92.4 pg/ml, where the sensitivity was 95.0% and specificity was 85.0% (Figure 6).

Higher serum levels of TLR4 in patients of Group II and Group III during all observation periods confirmed the functional activity and significance of the marker as a pro-inflammatory cytokine.

## Discussion

According to the recent research, acute tissue injury, caused by a thermal, chemical, or mechanical agent, results in a complex cascade of immune and inflammatory interactions (34). The expression of TLRs on antigen-presenting cells leads to further naive T cell priming and B cell activation, followed by pathogen recognition with the induction of adaptive immune responses (35). Continuous or excessive TLR4 activation or dysregulation of TLR4 signaling result in hyperproduction of pro-inflammatory mediators and can be accompanied by inflammatory and autoimmune diseases, including sepsis, atherosclerosis, rheumatoid arthritis, neuropathic pain, and neurodegenerative diseases (36). Watkins et al. have found that opioid agonists act as TLR4 agonists, while opioid antagonists (naltrexone, naloxone) act as TLR4 antagonists (37).

Exposure to morphine (or any other μ-opioids) can result in paradoxical hyperalgesia (38). Bai et al. suggest this phenomenon to be associated with TLR4 level as in case of normal TLR4 levels, minimal hyperalgesia signs are observed (39). Opioids can activate TLR4 on glial cells, by mimicking the interface of lipopolysaccharide (LPS), bind to TLR4 co-receptor MD-2 as a LPS analog, for direct activation of TLR4 (40). Wang et al. have found that morphine, similar to LPS, induces TLR4 dimerization and leads to the formation of the (TLR4/MD-2)/(TLR4/MD-2) heterotetramer. TLR4 and MD-2 have been found to be crucial for morphine-induced TLR4 pathway activation, as the production of nuclear factor kappa-light-chain-enhancer of activated B cells (NF-κB), IL-1β and TNF-α reduces, followed by the suppression of the peripheral and central immune systems (41). According to Takeda et al. TLR4 affect the induction, conversion, and maintenance of chronic pain (42). Complete tissue healing is necessary to stop the pain signaling process. However, if nociceptive stimuli persist, pathophysiological changes occurring at the peripheral, spinal, and supraspinal levels can cause chronic pain (43). Increasing evidence suggests the involvement of the immune system, including TLR4, in the development of chronic pain syndrome (44).

Moderate and severe pain affect more than 1.7 million children. Chronic primary pain is characterized by significant emotional or functional disability, while secondary pain is of a clear underlying etiology (disease, injury, nerve lesion or their treatment, e.g., surgery, chemotherapy, radiation therapy) (45). The prevalence rate for chronic pain in children ranges from 25 to 30% (46). Inadequate pain management at an early age affects the frequency, severity, and duration of chronic pain with subsequent maladaptive neurological changes in adulthood.

The results of this study confirmed the data on opioid-induced hyperalgesia and developing chronic pain in patients receiving conventional anesthesia management (Group III) and having high initial level of TLR4 at a discharge from the hospital already (44, 47–50).

## Conclusion

The use of regional anesthesia techniques was found to be accompanied by the minimum increase in pro-inflammatory marker TLR4 3 and 6 months after hospital discharge, thereby minimizing the development of chronic pain in children as compared to conventional anesthesia management (*p* < 0.05).

The advantages of the transversalis fascia plane block combined with the quadratus lumborum block (QLB + TFPB) *via* a single intramuscular injection are as follows: a significant effect on the level of pro-inflammatory marker TLR4 3 and 6 months after hospital discharge, ease of use, adequate perioperative pain management, reduced perioperative use of opioid analgesics and non-steroidal anti-inflammatory drugs, shortened length of hospital stay.

## Data availability statement

The original contributions presented in the study are included in the article/supplementary material, further inquiries can be directed to the corresponding author/s.

## Ethics statement

The article was approved by Ethics Ivano-Frankivsk Medical University LEC, protocol N°2/2022. Written informed consent was obtained from the individual(s), and minor(s)' legal guardian/next of kin, for the publication of any potentially identifiable images or data included in this article.

## Author contributions

All authors contributed to the production of this manuscript and have approved the final version.

## Conflict of interest

The authors declare that the research was conducted in the absence of any commercial or financial relationships that could be construed as a potential conflict of interest.

## Publisher's note

All claims expressed in this article are solely those of the authors and do not necessarily represent those of their affiliated organizations, or those of the publisher, the editors and the reviewers. Any product that may be evaluated in this article, or claim that may be made by its manufacturer, is not guaranteed or endorsed by the publisher.
